# Anti-*N*-methyl-D-aspartate receptor encephalitis presenting with acute psychosis in a preteenage girl: a case report

**DOI:** 10.1186/1752-1947-6-224

**Published:** 2012-07-30

**Authors:** Paraskevi Maggina, Mersini Mavrikou, Stavroula Karagianni, Chrysanthi L Skevaki, Antigoni Triantafyllidou, Constantinos Voudris, Eustathia Katsarou, Lela Stamogiannou, Sotiria Mastroyianni

**Affiliations:** 1 1st Department of Pediatrics, "P & A Kyriakou" Children’s Hospital, Athens, Greece; 2 Department of Pedopsychiatric Care, "P & A Kyriakou" Children’s Hospital, Athens, Greece; 3 Research Laboratories, "P & A Kyriakou" Children's Hospital, Athens, Greece; 4 Department of Neurology, "P & A Kyriakou" Children’s Hospital, Athens, Greece

**Keywords:** NMDAR, Encephalitis, Psychosis, Children

## Abstract

**Introduction:**

Anti-*N*-methyl-D-aspartate receptor (anti-NMDAR) encephalitis is a rare, newly defined autoimmune clinical entity that presents with atypical clinical manifestations. Most patients with anti-*N*-methyl-D-aspartate receptor encephalitis develop a progressive illness from psychosis into a state of unresponsiveness, with catatonic features often associated with abnormal movements and autonomic instability. This is the first report of anti-*N*-methyl-D-aspartate receptor encephalitis in a Greek pediatric hospital.

**Case presentation:**

An 11-year-old Greek girl presented with clinical manifestations of acute psychosis. The differential diagnosis included viral encephalitis. The presence of a tumor usually an ovarian teratoma, a common clinical finding in many patients, was excluded. Early diagnosis and prompt immunotherapy resulted in full recovery up to one year after the initial diagnosis.

**Conclusion:**

Acute psychosis is a rare psychiatric presentation in children, diagnosed only after possible organic syndromes that mimic acute psychosis are excluded, including anti-*N*-methyl-D-aspartate receptor receptor encephalitis. Pediatricians, neurologists and psychiatrists should consider this rare clinical syndrome, in order to make an early diagnosis and instigate appropriate treatment to maximize neurological recovery.

## Introduction

Anti-N-methyl-D-aspartate receptor (anti-NMDAR) encephalitis belongs to the autoimmune and paraneoplastic encephalitides [1]. Initial reports of a syndrome characterized by psychiatric manifestations, memory loss, decreased consciousness, and hypoventilation in four young women with ovarian teratomas were published in 2005 [2]. In 2007, auto-antibodies against the NMDAR were detected in these women and eight others with similar symptoms, seven of whom also had ovarian teratomas [2]. Most patients exhibit psychosis, memory loss, seizures, and language disintegration, and may develop unresponsiveness with catatonic features or periods characterized by abnormal movements, including autonomic and breathing instability [2]. Anti-NMDAR encephalitis mainly affects children and young adults and may be associated with malignancy. The presence of a tumor (usually an ovarian teratoma) is dependent on age, gender, and ethnicity, and is associated with a better prognosis after tumor resection and immunotherapy (corticosteroids, intravenous immunoglobulin, plasma exchange) than in patients who do not have tumors. More than 75% of all patients recover in association with declining antibody titers but relapses are frequent and a close follow-up is necessary, at least for two years, after initial diagnosis [2].

In this report, we present a case of anti-NMDAR encephalitis that was diagnosed in an 11-year-old Greek girl who presented with subacute encephalopathy and psychiatric manifestations without evidence of malignancy. To the best of our knowledge, this is the first pediatric patient diagnosed with anti-NMDAR encephalitis in Greece.

## Case presentation

A previously healthy 11-year-old girl with alternating confusion and agitation was admitted to the emergency department. She had a three day history of headaches, nausea, and sleepiness, 12 days prior to presentation at the hospital. Behavioral changes included low school performance, reduced food intake, troubled sleep, excessive and unintelligible speech referring mainly to her clothes, and panic possibly due to visual hallucinations, which intensified the day before admission. One week earlier, cranial magnetic resonance imaging (MRI) at another hospital was normal and she was diagnosed with an acute psychotic episode and prescribed olanzapine.

Neurological examination on admission noted stupor with agitation, fluctuation in consciousness with loss of ability to speak or make eye contact, limb dyskinesias, urinary incontinence, and self-harming behaviour (biting lips, knocking her head and her arms on the bed) (Table 1). The first days of hospitalization featured short periods of appropriate communication interrupted by behaviour indicating visual and auditory hallucinations. More frequent and intense episodes of agitation were followed by extrapyramidal signs and symptoms, including dystonia of the left leg and lower jaw movements, rhythmic tongue thrusting, chewing movements, teeth grinding, and lip biting. Seizures, mainly focal right or left sided convulsions were noted. Some of these episodes featured a truncal urticaria-like rash that receded in a few minutes and mild pyrexia (37 to 37.7^o^C). She was hemodynamically stable, with normal respiratory function.

**Table 1:** Patient’s characteristics and clinical manifestations, diagnostic findings and treatment

	**Patient’s characteristics and clinical manifestations**
**Sex**	female
**Age (years)**	11
**Prodromal symptoms**	yes
**Symptom presentation**	
** Behaviour/personality change**	yes
** Seizures**	yes
** Dystonia**	yes
** Orolingual dyskinesias**	yes
** Speech reduction**	yes
** Dystonic postures**	yes
** Muscle rigidity**	yes
** Autonomic instability**	yes (urinary incontinence)
** Central hypoventilation**	no
**MRI-abnormal findings**	no
**EEG-disorganized activity, focal or diffuse (non specific)**	yes
**EEG-epileptic activity**	no
**CSF-pleocytosis**	yes (5WBC/μL)
**CSF protein concentration >45mg/dl**	no
**CSF-oligoclonal bands**	yes
**Tumor-ovarian teratoma**	no
**Treatment**	
** Supportive care**	yes
** IVIG**	yes
** Glucocorticosteroids**	yes
** Cyclophosphamide**	yes
**Outcome**	
** Substantial improvement/Full recovery**	yes

*CSF* cerebrospinal fluid, *EEG* electroencephalography, *IVIG* intravenous immunoglobulin, *WBC* white blood cells, *MRI* magnetic resonance imaging.

Extensive investigations for metabolic, infectious, toxic, autoimmune, central nervous system and psychiatric disorders were performed. An electroencephalogram (EEG) demonstrated right-side slowing without epileptiform discharges. Cranial MRI was normal. Her cerebrospinal fluid (CSF) examination was normal including polymerase chain reaction (PCR) for herpes simplex viruses (HSV-1,2) and negative serology for viruses (adenovirus, enterovirus, cytomegalovirus, measles and rubella), toxoplasma, and mycoplasma, but oligoclonal bands were demonstrated. Toxicology tests on blood and urine were negative. No evidence was found for possible metabolic causes, including Wilson’s disease and porphyria. Thyroid function was normal. Negative anti-nuclear antibodies, antiphospholipid antibody, normal titer of anti-deoxyribonucleic acid (DNA) antibody to native DNA and absence of antibody to Sm nuclear antigen excluded systemic lupus erythematosus. Serum immunoglobulin M (IgM) for human herpes virus 1, 2, 6, Epstein-Barr virus, cytomegalovirus, enterovirus, adenovirus, measles, mumps, rubella, chickenpox, parvovirus B19, borrelia, mycoplasma and toxoplasma was negative. Anti-NMDAR antibodies were revealed on immunohistochemistry in her serum and CSF. The association of anti-NMDAR antibodies with ovarian teratoma and other abdominal and thoracic tumors prompted pelvic ultrasonography, abdominal MRI and thoracic X-ray computed tomography (CT), all of which were normal. Testing for related tumor markers (alpha-fetal protein [α-FP], and human chorionic gonadotropin [β-hCG]) was negative.

Initial intervention was mostly symptomatic treatment of acute psychosis including diazepam (5–7.5mg/day IV), fluoxetine (10mg/day orally), risperidone (0.5mg orally 3 times per day) and biperiden (2mg orally 1 to 2 times per day). During severe episodes of agitation, she received midazolam (0.1mg/kg/dose IV).

Anti-NMDAR encephalitis was diagnosed 16 days after admission, and intravenous immunoglobulin (0.4g/kg every 24 hours for five days) and pulses of methylprednisolone (30mg/kg for five doses) were administered followed by oral prednisolone with progressive tapering of the dose. She also received three pulses of cyclophosphamide (750mg/m2), the first pulse 32 days after admission, and then at monthly intervals.

Symptoms improved following immunotherapy with steroids and intravenous immunoglobulin, and the frequency and duration of episodes of agitation and dystonia declined. The need for sedation medication (midazolam intravenous) was substantially reduced and discontinued after the last episode of agitation, on day 33. Fluoxetine was discontinued after the diagnosis of anti-NMDAR encephalitis; risperidone and diazepam were gradually withdrawn four weeks later. Communication and mobility improved 15 days after the administration of the first pulse of cyclophosphamide, and she was discharged on day 71 with mild dystonic gait and significant psychiatric manifestations; her verbal communication was poor, she had recent memory deficits, but she was able to read and make simple calculations. She demonstrated progressive improvement of cognitive and motor neurological functions over the next six months. She recovered fully and returned to school without apparent cognitive or behavioral problems after one year. Repeat testing for anti-NMDAR antibodies in her blood, five months after diagnosis, was negative and the EEG results were normal.

## Discussion

This clinical case presentation with symptoms resembling acute psychosis is consistent with the diagnosis of anti-NMDAR encephalitis, an autoimmune disorder described recently in young women with ovarian teratoma [3,4]. The autoimmune pathogenesis [5] is mediated by antibodies, detected in serum and CSF, that mainly target the NR1 subunit of the NMDA receptor on the cell surface of neurons [3,6].

It has been demonstrated that this disorder is more frequent in children than previously thought, and that clinical manifestations in pediatric patients are similar to those of adults [7]. Prodromal symptoms such as low fever, headache, common cold-like symptoms, and gastroenteritis are found in most cases. The most frequent clinical presentation resembles acute psychosis [5] (anxiety, agitation, paranoia, visual and auditory hallucinations), but recognizing such symptoms in young children may be difficult. Dystonia and dyskinesias, abnormal movements of the face, tongue, limbs, trunk, and abdomen are common. Seizures can be noted although not demonstrated by EEG and there must be caution about any excessive use of antiepileptic drugs in these patients. Autonomic manifestations such as urinary incontinence and sleep dysfunction are common. However, more severe autonomic dysregulation such as central hypoventilation, dysrhythmia, tachycardia, and hyperthermia is rarely seen in children.

In children the frequency of teratoma [3] is low compared to that in adults. However, some tumors are detected many months after the initial encephalitic episode. Since some investigations have age-related limitations, all patients should undergo ultrasound and MRI of the abdomen and pelvis at initial diagnosis, as well as periodically for at least two years after the first episode.

Treatment includes surgical removal of ovarian teratoma or other tumors and prompt and aggressive immunotherapy [2,8]. Some children appear to respond rapidly to intravenous immunoglobulin and methylprednisolone but may exhibit slow improvement independent of the treatment used. Other therapeutic options include cyclophosphamide, anti-CD20 monoclonal antibodies (rituximab), and plasma exchange, mainly in more severe/persistent cases. Studies published to date indicate a favorable prognosis for most young patients (75% respond to initial treatment, 29% recover fully, 45% improve significantly, and 26% reveal minor improvement) [7]. The outcome improves following removal of a teratoma, if present, in combination with immunotherapy [9,10]. Relapses have been noted in approximately 25% of young patients and seem to occur while tapering immunotherapy, as well as in cases without teratoma in those with high titers of anti-NMDAR antibodies in the CSF. Follow-up evaluation [7] should include neurologic and psychiatric examinations, ultrasound and MRI of the pelvis and abdomen, and measurements of the levels of anti-NMDAR antibodies’ titers in serum and CSF.

## Conclusion

The diagnosis of ‘acute psychosis’ in children is rare and can be made only after possible organic causes are excluded. The differential diagnosis is broad, with viral encephalitis being the most likely alternative cause in these circumstances. Anti-NMDAR encephalitis is a new autoimmune disorder that commonly presents with psychiatric manifestations. Pediatricians, neurologists and psychiatrists should be aware of this disease’s clinical spectrum to identify and properly treat these patients.

Once specific anti-NMDAR antibodies are detected, it is crucial to start correct treatment, including tumor removal and intense immunotherapy, immediately. This disease has a good prognosis in the majority of cases if diagnosed early and treated properly.

## Abbreviations

CSF, cerebrospinal fluid; EEG, electroencephalogram; HSV, herpes simplex virus; IVIG, intravenous immunoglobulin; MRI, Magnetic resonance imaging; NMDAR, N-methyl-d-aspartate-receptor; NR1, NMDA receptor 1; PCR, polymerase chain reaction; α-FP, α-fetoprotein; β-hCG, β-human horionic gonadotropin.

## Consent

Written informed consent was obtained from the patient’s parents for publication of this manuscript and accompanying images. A copy of the written consent is available for review by the Editor-in-Chief of this journal.

## Competing interests

The authors declare that they have no competing interests.

## Authors’ contributions

PM and SM analyzed the data and wrote the manuscript. PM, MM**,** SK, AT, CV, EK, LS and SM were involved in the initial diagnosis and management. CLS was involved in the process of drafting and revising the manuscript. All authors have read and approved the final manuscript.
